# Colds Cured

**Published:** 1859-11

**Authors:** 


### COLDS CURED.

It would be to the saving of human health and happiness, and life itself, if the periodical press would never publish a recipe for any human ailment, which involved the taking of anything into the stomach.

Some scrap-editor characterizes it as an excellent remedy for a cough caused by a common cold, to soak an unbroken egg for forty-eight hours in half a pint of vinegar, then add as much honey, break up all together, and take a teaspoonful for a dose several times a day.

If the writer of that recipe had possessed the smallest amount of common observation, he would have known that if a man begins to cough, as the result of a common cold, it is the result of nature herself attempting the cure, and she will effect it in her own time, and more effectually than any man can do, if she is only let alone, and her instincts cherished. What are those instincts? She abhors food, and craves warmth. Hence, the moment a man is satisfied that he has taken a cold, let him do three things: 1st, eat not an atom; 2d, go to bed and cover up warm in a warm room; 3d, drink as much cold water as he wants, or as much hot herb tea as he can, and in three cases out of four, he will be almost entirely well within thirty-six hours.

If he does nothing for his cold for forty-eight hours after the cough commences, there is nothing that he can swallow that will, by any possibility, do him any good, for the cold, with such a start, will run its course of about a fortnight, in spite of all that can be done, and what is swallowed in the meantime, in the way of physic, is a hindrance and not a good.

"*Feed a cold and starve a fever*," is a mischievous fallacy. A cold always brings a fever; the cold never begins to get well until the fever begins to subside; but every mouthful swallowed is that much more fuel to feed the fever, and, but for the fact that as soon as the cold is fairly seated, nature, in a kind of desperation, steps in and takes away the appetite, the commonest cold would be followed by very serious results, and in frail people, would be almost always fatal.

These things being so, the very fact of waiting forty-eight hours, gives time for the cold to fix itself in the system, for a cold does not usually cause cough until a day or two has passed, and then to wait two days longer, gives it its fullest chance to do its work before anything at all is done.

